# Butyrylcholinesterase distribution in the mouse gastrointestinal tract: An immunohistochemical study

**DOI:** 10.1111/joa.13754

**Published:** 2022-08-25

**Authors:** Ilenia Severi, Silvia Abbatelli, Jessica Perugini, Eleonora Di Mercurio, Martina Senzacqua, Antonio Giordano

**Affiliations:** ^1^ Department of Experimental and Clinical Medicine Marche Polytechnic University Ancona Italy

**Keywords:** acetylcholine, non‐neuronal cholinergic system, parietal cells, Paneth cells, Ki67, ghrelin, acetylcholinesterase

## Abstract

Butyrylcholinesterase (BChE) is a hydrolytic enzyme that together with acetylcholinesterase (AChE) belongs to the cholinesterase family. Whereas AChE has a well‐established role in regulating cholinergic neurotransmission in central and peripheral synapses, the physiological role of BChE remains elusive. In this morphological immunohistochemical and double‐label confocal microscopy study we investigated the distribution of BChE in the mouse gastrointestinal tract. BChE‐positive cells were detected in the liver (both in hepatocytes and cholangiocytes), in the keratinised layers of the squamous epithelium of the oesophagus and forestomach, in the oxyntic mucosa of the stomach, in the mucus‐secreting cells of duodenal Brunner glands and the small and large intestinal mucosa. Interestingly, BChE‐positive cells were often detected close to gastrointestinal proliferative niches. In the oxyntic mucosa, the close proximity of ghrelin‐producing and BChE‐positive parietal cells suggests that BChE may be involved in ghrelin hydrolysation through paracrine action. To our knowledge, this is the first comprehensive morphological study performed to gain insight into the physiological role of BChE in the gastrointestinal tract.

## Graphical abstract

Representative confocal microscopy images of the glandular stomach showing BChE+ cells that also express H+,K+‐ATPase, a widely used marker of mouse gastric parietal cells.

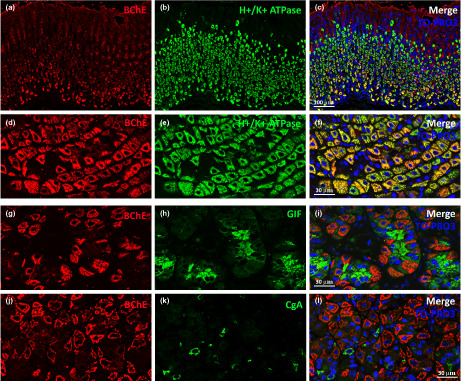

## 1 INTRODUCTION

Acetylcholine (ACh) is an exemplary neurotransmitter prototype. In the early decades of the 20th century, the study of its action on the frog heartbeat allowed establishing the chemical nature of neural transmission (Dale, 1914; Loewi, 1921). In neurons, it is synthesised from choline and acetyl‐coenzyme A by choline acetyltransferase (ChAT), then stored in presynaptic vesicles and released in the synaptic cleft upon arrival of the action potential. From here, it reaches the postsynaptic membrane, where it can bind to muscarinic and/or nicotinic receptors (Maurer & Williams, 2017).

Neurons are not the only cells capable of synthesising ACh. Notably, non‐neuronal cells including keratinocytes, bronchial cells, endothelial cells and immune cells express ChAT and can both synthesise and release ACh (Reichrath et al., 2016; Wessler et al., 1998). Its widespread synthesis, coupled with the almost ubiquitous expression of ACh receptors in all organs (including the human placenta, which is devoid of autonomic innervation; Sastry, 1997) suggests that mammals are endowed with non‐neuronal cholinergic systems that are characterised by multiple albeit poorly characterised physiological functions (Reichrath et al., 2016). However, in mammalian organs this powerful molecule needs to be confined to the area where it is released, to prevent noxious hormone‐like actions. This posits the existence of an enzyme system capable of its rapid hydrolysation.

Mammals possess two cholinesterases, acetylcholinesterase (AChE, EC. 3.1.1.7) and butyrylcholinesterase (BChE, EC. 3.1.1.8), the latter also called pseudo or non‐specific cholinesterase (Massoulié et al., 1993). Whereas AChE is primarily found in the central and peripheral nervous systems, where it underpins neuronal transmission at cholinergic synapses, BChE is also found in several peripheral organs including the lung, heart, kidney and liver (Massoulié et al., 1993). BChE expression is especially high in the liver, which is the main source of plasma BChE (Jbilo et al., 1994). Together, AChE and BChE activities determine and regulate the action of ACh in peripheral organs and the central nervous system (Vaknine & Soreq, 2020).

BChE is considered a non‐specific cholinesterase due to its ability to hydrolyse as substrates for other choline and non‐choline esters (Massoulié et al., 1993). However, unlike AChE, its physiological function is still debated and numerous hypotheses have been advanced.

As a bioscavenger molecule, BChE appears to be one of the enzymes involved in the detoxification of organophosphates, drugs and dietary compounds (Hou et al., 2013; Johnson & Moore, 2012). Moreover, plasma and liver BChE activity are elevated in patients with obesity and diabetes and rodent models of metabolic disorders (Chen et al., 2017; Perelman et al., 1990; Randell et al., 2005), whereas it is low in individuals suffering from malnutrition or inflammatory states (Lampon et al., 2012). Despite the clinical importance of these discoveries, few studies have tried to establish a causal relationship between BChE and obesity. Findings from two recent studies point to a role for it in hydrolysing ghrelin, the appetite‐promoting hormone produced by the stomach (Brimijoin et al., 2016; Chen et al., 2017). These studies have found that BChE hydrolyses ghrelin at a rate that strongly affects weight gain and fat metabolism (Brimijoin et al., 2016) and that BChE knockout mice fed a high‐fat diet rapidly become obese and show 50% higher plasma ghrelin levels (Chen et al., 2017).

The role of BChE in ghrelin regulation and its altered expression in patients with metabolic dysfunction suggests that it plays a physiological role in the gastrointestinal (GI) tract. Evidence for its distribution in the GI tract mostly comes from biochemical studies; of these, few have examined its presence in the rodent and human stomach and intestine (Cheng et al., 2008; L'Hermite et al., 1996; Roivainen et al., 2004; Sine et al., 1988, 1991; Wang et al., 2004).

In this study, we investigated the presence and distribution of BChE in the mouse GI tract using peroxidase immunohistochemistry and confocal microscopy.

Collectively, our findings show that BChE is widely expressed in epithelial secretory cells; in particular, BChE‐positive (^+^) cells were detected in the keratinised epithelium of the oesophagus and the forestomach, in the glandular portion of the stomach and the small and large intestinal epithelium. This distribution suggests a dual role for BChE against the compounds passing through the gastric and intestinal lumina and/or in the physiological processes that take place in the mucosal layer of the GI tract.

## 2 MATERIALS AND METHODS

### 2.1 Animals and tissue processing

Adult male C57BL/6 mice purchased from Charles River Laboratories (Calco, Italy) were housed in plastic cages in constant environmental conditions in a 12 h light/dark cycle at 22°C with free access to standard chow and water. Handling was limited to cage cleaning. Their care was according to Council Directive 2010/63/EU. All experiments were approved by the Italian Health Ministry (Authorisation no. 405/2018‐PR). After a week on the normal diet, animals were divided into 2 groups of 6 with similar body weight and fed for 10 weeks either the normal diet (Control group) or a high‐fat diet (HFD; Charles River; 50 kJ% from fat, 30 kJ% from carbohydrates and 20 kJ% from proteins; HFD group). At 12–14 weeks all mice were sacrificed. The livers of both mouse groups were analysed to test the specificity of the primary BChE antibody. All the other analyses were performed only in Control mice.

The animals used for morphological analyses were anaesthetised with an overdose of 2,2,2‐tribromoethanol (Sigma–Aldrich) and perfused transcardially with 4% paraformaldehyde in 0.1 M phosphate buffer (PB), pH 7.4. The oesophagus, stomach, small and large intestine and liver were dissected and postfixed with the same fixative solution for 24 h at 4°C before being washed in PB. Mice used for western blotting were anaesthetised and euthanised by cervical dislocation; the livers were rapidly removed, snap‐frozen in liquid nitrogen and stored at −80°C until use.

### 2.2 Antibodies

The primary and secondary antibodies used in the study are listed in Table 1.

**TABLE 1:** Antibodies used in the study.

Antibodies	Host	Dilution	Source
(a) Primary antibodies			
Butyrylcholinesterase (BChE)	G	1:1000 (IHC), 1:400 (IF), 1:500 (WB)	AF‐9024, R&D Systems, Minneapolis, MN, USA (lot no. CDJH0117111)
Ghrelin	R	1:400 (IF)	MAB‐8200, R&D Systems
H+/K+‐ATPase	M	1:1000 (IF)	sc‐374094, Santa Cruz Biotech (Santa Cruz, CA, USA)
Gastric intrinsic factor (GIF)	Ra	1:1500 (IF)	PA5‐87282, Invitrogen, Thermo Fisher Scientific, MA, USA
Chromogranin‐A (Chr‐A)	M	1: 400 (IF)	sc‐393941, Santa Cruz Biotech
Lysozyme	Ra	1:80 (IF)	MA5‐32154, Invitrogen
Olfm4 (D6Y5A) XP	Ra	1:150 (IF)	39141; Cell Signaling, Danvers, MA, USA
β‐actin	M	1:200 (WB)	Sc‐477778, Santa Cruz Biotech

Host	Application	Dilution	Source
(b) Secondary antibodies			
Anti‐goat IgG, Alexa Fuor^TM^ 488	IF	1:400	A11055, Invitrogen, Thermo Fisher Scientific, MA, USA
Anti‐mouse IgG, Alexa Fuor^TM^ 488	IF	1:400	A21202, Invitrogen
Anti‐rabbit IgG, Alexa Fuor^TM^ 488	IF	1:400	A21206, Invitrogen
Anti‐rat IgG, Alexa Fuor^TM^ 488	IF	1:400	A21208, Invitrogen
Anti‐mouse IgG, Alexa Fuor^TM^ 555	IF	1:400	A31357, Invitrogen
Anti‐goat IgG, Alexa Fuor^TM^ 555	IF	1:400	A21432, Invitrogen
Anti‐goat IgG, Peroxidase‐labelled	WB	1:1000	PI‐9500, Invitrogen,
Anti‐mouse IgG, peroxidase‐labelled	WB	1:5000	715‐036‐150, Jackson ImmunoResearch, Cambridge, UK
Anti‐goat IgG, biotinylated	IHC	1:200	BA‐5000, Invitrogen

Abbreviations: G, goat; IF, immunofluorescence; IF, immunofluorescence; IHC, immunohistochemistry; IHC, immunohistochemistry; M, mouse; R, rat; Ra, rabbit; WB, Western blotting; WB, Western blotting.

### 2.3 Western blotting

Liver tissue lysates were obtained by homogenisation in RIPA buffer (150 mM NaCl, 10 mM Tris, pH 7.2, 0.1% SDS, 1.0% Triton X‐100, 5 mM EDTA, pH 8.0) containing a protease inhibitor cocktail (Roche Applied Science). Samples were centrifuged and protein concentrations were determined by the Bradford assay (Bio‐Rad Laboratories). Total protein extracts (20–15 μg) were separated by 10% SDS‐PAGE and transferred to a nitrocellulose membrane using Bio‐Rad's Trans‐Blot Turbo TM Transfer system. Loading and transfer efficiency were checked by Ponceau staining (Santa Cruz Biotechnology). Membranes were then blocked for 1 h at room temperature (RT) in TBS‐Tween 20 containing 5% non‐fat dried milk and subsequently incubated overnight with primary antibodies (Table 1a). Finally, they were incubated with a secondary antibody conjugated to horseradish peroxidase (Vector Laboratories; Table 1b) for 1 h at RT. Bands were visualised with the Chemidoc Imaging System using the Clarity™ Western ECL chemiluminescent substrate (all from Bio‐Rad). Densitometric analysis was performed with Bio‐Rad's Image Lab software.

### 2.4 Peroxidase immunohistochemistry

Serial paraffin sections (3 μm thick) were obtained with a PFM slide microtome (Bio‐Optica), dried and stored until use. In brief, after dewaxing, paraffin sections were reacted with H_2_O_2_ (3% in deionised H_2_O; 5 min) to block endogenous peroxidase, rinsed twice in phosphate‐buffered saline (PBS) and treated with normal serum blocking solution (2% in PBS; 20 min). Next, they were incubated with the polyclonal goat anti‐BChE antibody (Table 1a). After a thorough rinse in PBS, sections were incubated in a 1:200 v/v biotinylated secondary antibody solution (in PBS; 30 min; anti‐goat IgG biotinylated, BA‐5000, Invitrogen, Thermo Fisher Scientific), rinsed in PBS and incubated in avidin‐biotin‐peroxidase complex (Vectastain Elite ABC Kit, Peroxidase, Vector Laboratories), washed several times in PBS and finally incubated in 3,3‐diaminobenzidine tetrahydrochloride (Impact DAB Substrate kit, Peroxidase, Vector Laboratories, 5 min). Sections were finally counterstained with haematoxylin, dehydrated, cleared with xylene and mounted in Eukitt medium (Sigma–Aldrich). Staining was never observed when the primary antibody was omitted.

### 2.5 Immunofluorescence and confocal microscopy

For immunofluorescence, paraffin sections were dewaxed, washed in 0.1% PBS‐Tween and incubated with an antigen retrieval solution (Histo‐VT‐One, PH 9, Nacalai Tesque) for 40 min at 70°C. After a thorough rinse in 0.1% PBS‐Tween, they were incubated with blocking solution (Blocking One Histo, Nacalai Tesque) at RT for 40 min. Next, they were incubated with a mixture of two primary antibodies diluted in 1% bovine serum albumin (BSA)‐PBS (for dilutions see Table 1a) and incubated overnight at 4°C. The next day sections were washed twice with 0.1% PBS‐Tween and incubated in a cocktail of fluorophore‐linked secondary antibodies (Alexa Fluor, Invitrogen; Table 1b) diluted 1:400 in 1% BSA‐PBS for 30 min at RT. Sections were subsequently washed twice with 0.1% PBS‐Tween, stained with TO‐PRO3, mounted on standard glass slides, air‐dried and coverslipped using Vectashield mounting medium (Vector Laboratories). Sections were viewed under a motorised Leica DM6000 microscope at different magnifications; fluorescence was detected with a Leica TCS‐SL spectral confocal microscope (both from Leica Microsystems, Wetzlar, Germany) equipped with an Argon and He/Ne mixed gas laser. Fluorophores were excited with the 488, 543 and 649 nm lines and imaged separately. Images (1024 × 1024 pixels) were obtained sequentially from two channels using a confocal pinhole of 1.1200 and stored as TIFF files. The brightness and contrast of the final images were adjusted using Photoshop 6 (Adobe Systems).

### 2.6 Statistical analysis

All values are mean ± standard error of the mean (SEM). Data were analysed for significance with GraphPad Prism (version 8). Differences between groups were analysed using an unpaired student's *t*‐test. The threshold for significance was *p* < 0.05.

## 3 RESULTS

### 3.1 Liver BChE expression and distribution in the liver from mice fed a normal diet or the HFD

**FIGURE 1:**
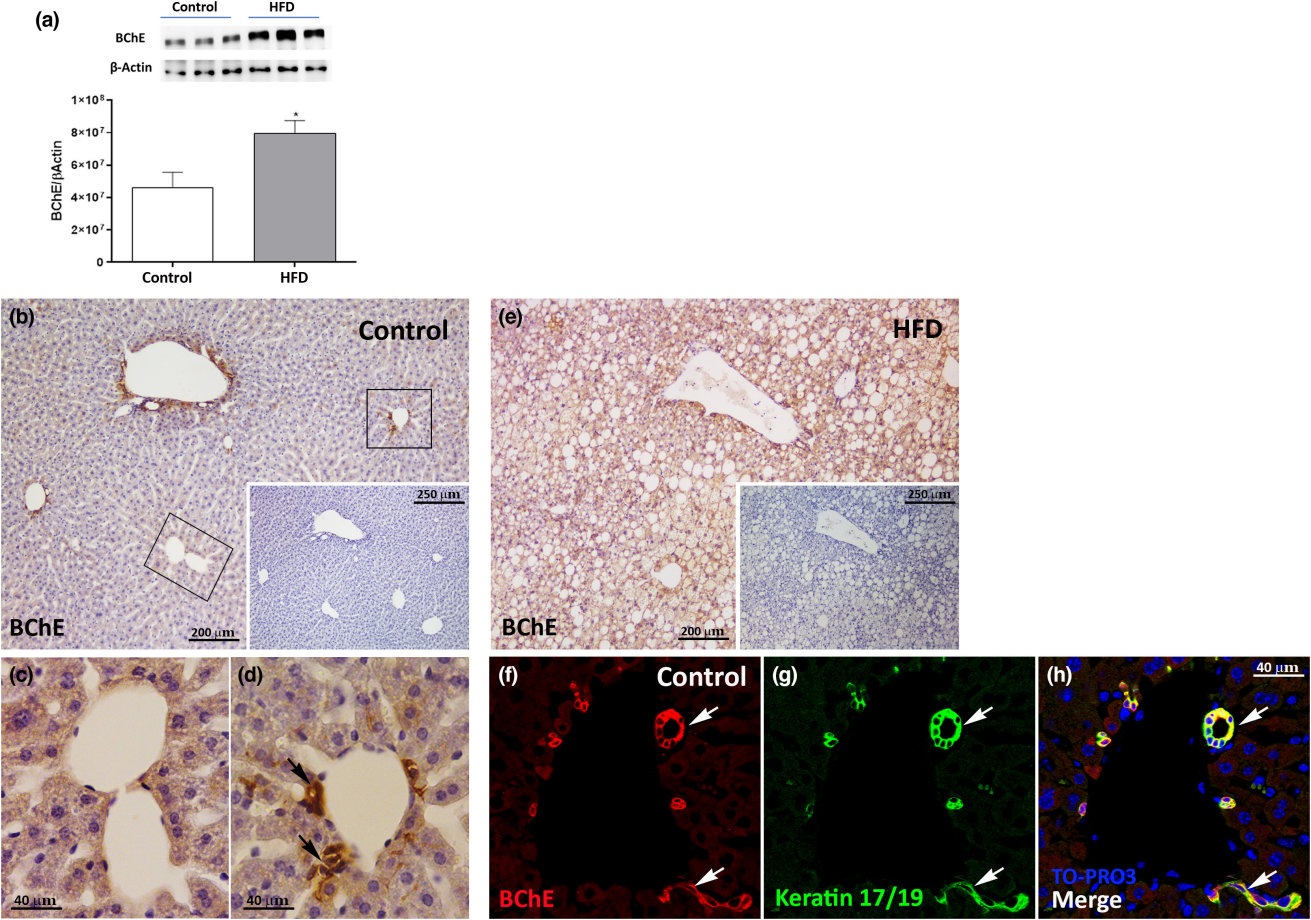
BChE expression and distribution in the liver from mice fed a normal or a high‐fat diet. (a) Representative immunoblot and BChE quantification in liver protein extract from the Control and the HFD groups. Data from 3 mice are expressed as mean ± SEM (student's *t*‐test). (b) Representative immunoperoxidase image showing BChE immunoreactivity in a liver section from a Control mouse. Specific BChE staining is visible in hepatocytes, enlarged in (c), and in epithelial cells lining intrahepatic biliary ducts, enlarged in (d). Inset on the bottom right corner in (a) negative control without the primary antibody. Dark arrows indicate epithelial cells lining BChE‐positive intrahepatic biliary ducts (e) Representative immunoperoxidase image showing BChE immunoreactivity in a liver section from an HFD mouse. Inset on bottom right corner: negative control without the primary antibody. (f–h) Representative confocal images from liver sections from a Control mouse showing BChE^+^ cells (red) that also express keratin 17/19, a cholangiocyte marker (green). White arrows indicate bile ducts positive for both keratin 17/19 and BChE.

Since BChE is mainly synthesised in the liver and its levels are abnormally high in plasma and liver of HFD mice (Chent e al., 2017), the specificity of the primary BChE antibody was tested in the liver samples from Control and HFD mice using Western blot analysis and immunohistochemistry. All the other morphological analyses of GI tract organs, involved in the study, were performed only in Control mice. We used an affinity‐purified goat polyclonal anti‐BChE antibody (Table 1a) that yields a single band of 75 kDa. As expected, BChE was significantly more abundant in the liver of HFD mice (Figure 1a, *n* = 3, *p* < 0.05). By immunohistochemistry, BChE^+^ hepatocytes were detected in the liver of Control mice (Figure 1b,c). In these mice, BChE positivity was also detected in scattered epithelial cells lining the intrahepatic biliary ducts (Figure 1d). Hepatocyte staining was more intense in HFD mice (Figure 1e), in line with a previous report (Chen et al., 2017). In Control mice, double‐label experiments showed that BChE^+^ cells near the portal capillaries, resembling biliary epithelial cells, were also positive for keratin 17/19, a cholangiocyte marker (Figure 1f–h). To our knowledge, this is the first report describing BChE^+^ cholangiocytes in mouse liver.

### 3.2 BChE distribution in the oesophagus

**FIGURE 2:**
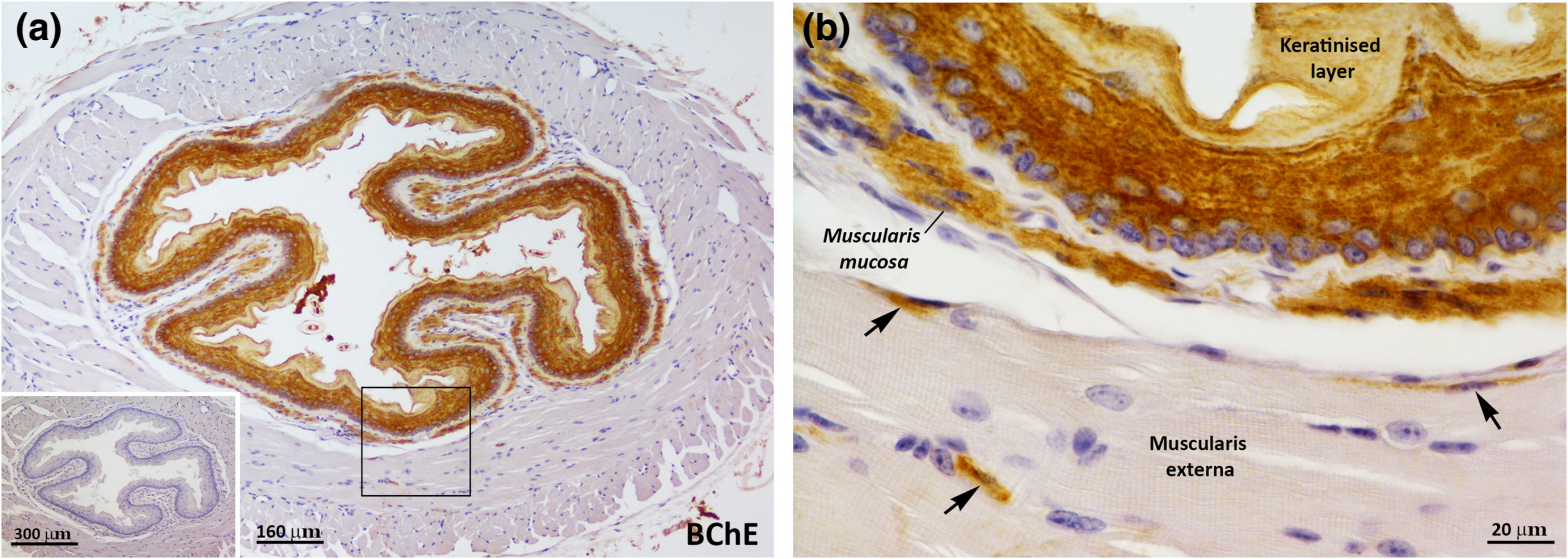
BChE distribution in mouse oesophagus. (a) Representative immunoperoxidase image showing BChE immunoreactivity in an oesophagus tissue section. Specific BChE staining is visible in the oesophageal epithelial layers, in the muscularis mucosa layer and the oesophageal lumen. Inset on the bottom left corner: negative control without the primary antibody. (b) Enlargement of the area framed in (a), showing BChE^+^ cells in the muscularis mucosa layer and scattered positive cells in the muscularis externa.

Immunohistochemical analysis disclosed that the cells of the 4‐6 layers of the keratinised squamous epithelium and the smooth muscle cells of the muscularis mucosa were positive for BChE (Figure 2a,b). Moreover, scattered BChE^+^ cells were detected in the muscular layers of the muscularis externa (Figure 2b). The elongated shape of these BChE^+^ cells is reminiscent of the endothelial cells or smooth muscle cells that are found in the muscularis externa of adult mouse oesophagus (Rishniw et al., 2007). In particular, these sections were near the stomach, in the mid‐lower portion of the oesophagus. Notably, BChE staining seemed to be found not only in the keratinised epithelial layer but also in the oesophageal lumen (Figure 2a), possibly as a result of the desquamation of keratinised cells and/or secretory processes.

### 3.3 BChE distribution in the stomach

The mouse stomach can be divided into forestomach, characterised by a keratinised squamous mucosa resembling the oesophageal mucosa, and glandular stomach, which in turn can be divided into the squamocolumnar junction, corpus and pyloric region (Ghoshal & Bal, 1989). At the junction of the forestomach and glandular stomach, the mucosa forms a “limiting ridge” (Luciano & Reale, 1992) covered by a thick, keratinised, stratified squamous epithelium. In the glandular stomach, the oxyntic glands of the corpus are clearly distinct from mucus‐secreting pyloric glands (Engevik et al., 2020).

**FIGURE 3:**
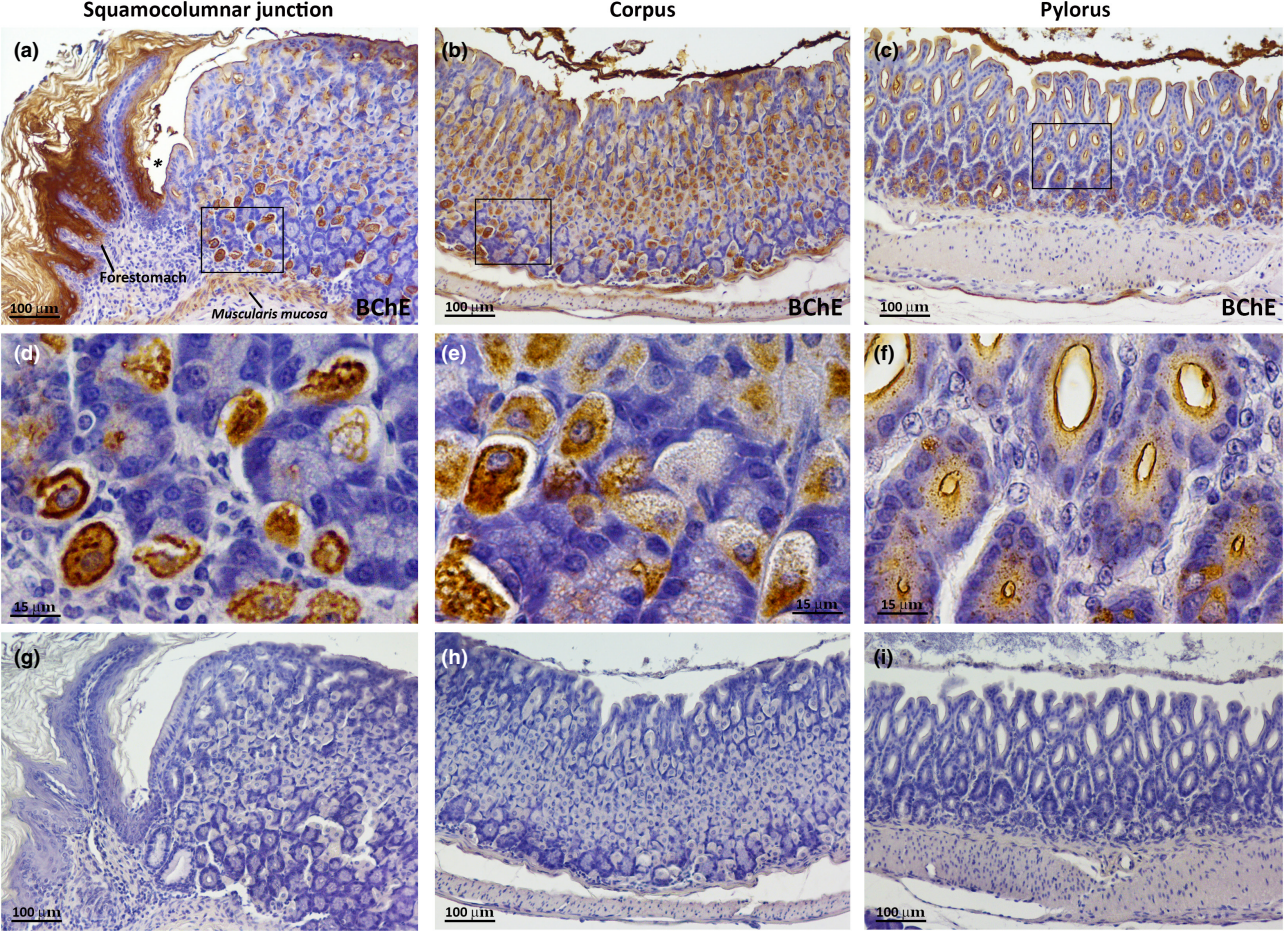
BChE distribution in the mouse stomach. (a–c) Representative immunoperoxidase images showing BChE immunoreactivity in the glandular stomach, specifically the squamocolumnar junction region (a), the corpus (b), and the pyloric region (c). Specific BChE staining is visible in the keratinised epithelial cells of the forestomach (a), in the short glands of the cardiac region (a), in the middle and lower portion of corpus glands (b) and in the pyloric region (c), where immunopositive cells decline markedly. (d–f) Enlargements of BChE^+^ cells located in the framed areas in a, b and c, respectively. (g–i) Negative controls without the primary antibody of the experiments shown in a, b and c.

BChE^+^ cells were detected in the keratinised epithelial cells of the forestomach; notably, their distribution pattern was similar to the one described in the oesophagus (Figure 3a). BChE^+^ cells were also identified in the short glands of the cardiac region near the limiting ridge; some of these cells, lying in the lower third of the mucosa (corresponding to the gland base), were intensely stained, whereas some BChE^+^ cells found in the middle portion of the mucosa (corresponding to the gland neck) displayed weaker staining (Figure 3a). BChE^+^ cells were most numerous in the corpus, where they were detected in the lower and middle portion of the gastric glands (Figure 3b), whilst they were much fewer in the pyloric region, which in mice consists mainly of mucous cells (Figure 3c). Interestingly, BChE^+^ gastric juice was detected in the glands and the apical portion of the gastric mucosa in all three glandular stomach regions (Figure 3a–c). Moreover, in all three regions, the smooth muscle cells of the muscularis mucosa were BChE^+^ (Figure 3a–c).

At high magnification, the BChE^+^ gland cells were large and oval, with central roundish nuclei and microvilli‐like structures (Figures 3d,e). Their morphology and distribution in the oxyntic mucosa of the stomach suggest that they may be parietal cells. Notably, oval parietal cells contain membranes that upon stimulation can fuse with the apical secretory canaliculi to form microvilli‐like structures, thus expanding their secretory surface (Engevik et al., 2020).

The gastric oxyntic glands are characterised by heterogeneous cell types, including parietal, chief and enteroendocrine cells. Chief and acid‐secreting parietal cells are found throughout the glands except in the pit region, whereas enteroendocrine cells are mainly found at the gland base (Willet & Mills, 2016).

**FIGURE 4:**
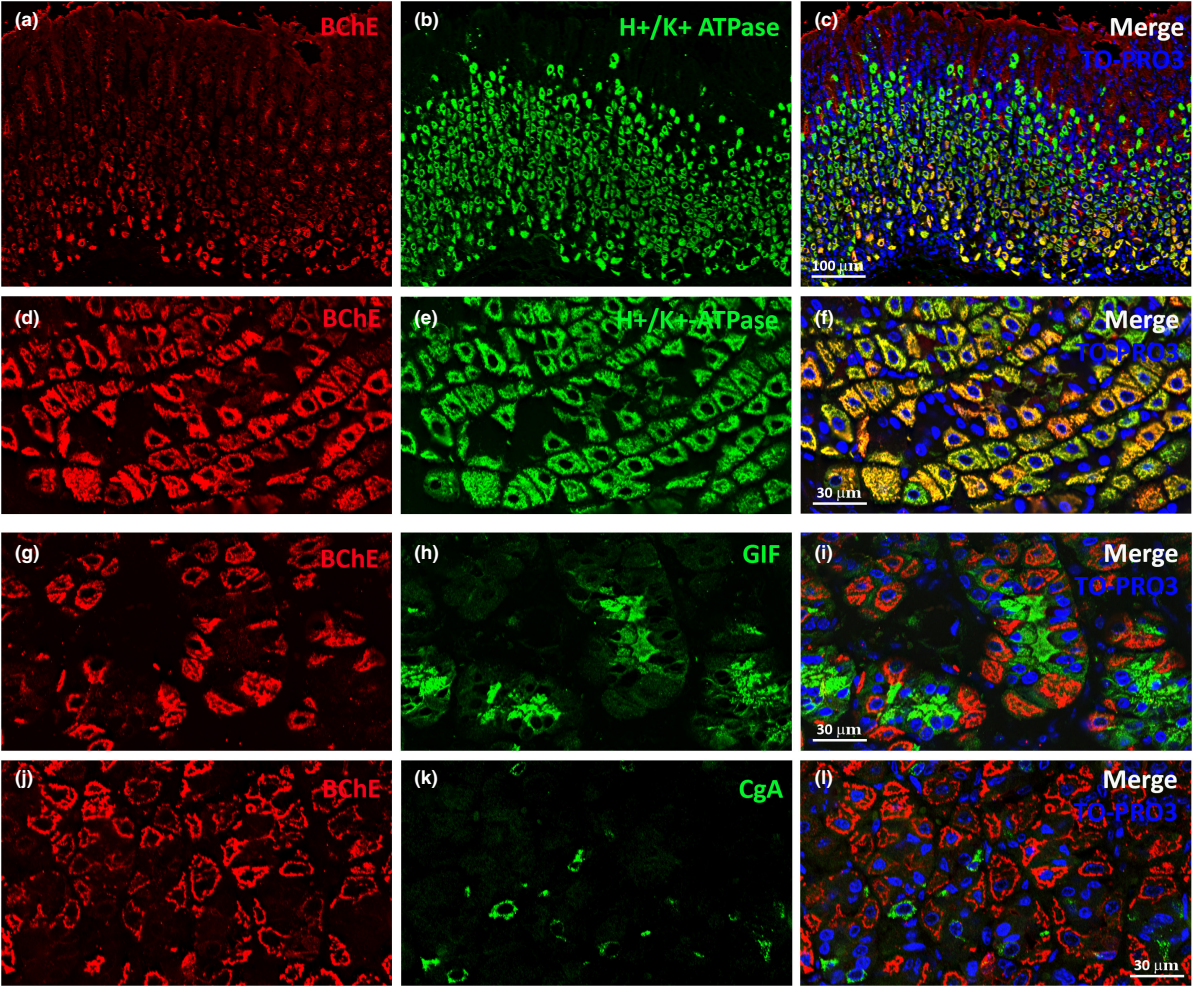
BChE^+^ mouse gastric cells. (a–c) Representative confocal microscopy images of the glandular stomach in the corpus region showing BChE^+^ cells (red) that also express H^+^,K^+^‐ATPase, a widely used marker of mouse gastric parietal cells (green). (d–f) At higher magnification, nearly all BChE^+^ cells (red) are also H^+^,K^+^‐ATPase‐positive (green). (g–i) Representative confocal images from the glandular stomach in the corpus region showing BChE^+^ cells (red) that are negative for, but very close, to cells positive for GIF, a widely used marker of mouse gastric chief cells (green). (j–l) Representative confocal images of the glandular stomach in the corpus region showing BChE^+^ cells (red) that are negative for, but in close proximity to, cells positive for CgA, a widely used marker of mouse gastric neuroendocrine cells (green).

Double‐label experiments, performed to confirm the phenotype of BChE^+^ cells in oxyntic glands, showed that 94.54 % ± 0,60 (*n* = 3) were positive for H^+^,K^+^‐ATPase, a common marker of gastric parietal cells (Figure 4a–c, enlarged in Figure 4d–f; Engevik et al., 2020). Some BChE^+^ cells negative for H^+^,K^+^‐ATPase were occasionally detected at the gland base.

In contrast, BChE^+^ cells in mouse oxyntic glands were negative for gastric intrinsic factor (GIF; Figure 4g–i) and chromogranin‐A (CgA; Figure 4j–l), respectively, a chief cell and an enteroendocrine cell marker (Boass & Wilson, 1964; Dieckgraefe et al., 1988; Engelstoft et al., 2015). GIF is a major secreted protein of human parietal cells which in mice and rats is found in zymogenic chief cells (Lorenz & Gordon, 1993), whereas CgA is an acidic protein found in large dense‐core secretory vesicles that are believed to be expressed in all cells of the mouse enteroendocrine GI tract (Engelstoft et al., 2015).

Since BChE plays a role in ghrelin hydrolysis by cleaving the octanoyl group (Brimijoin et al., 2016; Chen et al., 2017), double‐label immunofluorescence experiments were conducted to establish whether BChE^+^ cells also produce ghrelin. Although BChE^+^ cells were detected in close proximity to ghrelin‐producing cells in the corpus oxyntic mucosa, BChE staining was never observed in ghrelin‐producing cells (Figure S1, enlarged in Figure S1).

### 3.4 BChE distribution in the small intestine

The mouse small intestine can be divided into three functionally but not morphologically distinct units: duodenum, jejunum and ileum (McGraw‐Hill, 1966). Brunner's tubuloalveolar glands of the duodenum consist of pyramidal mucous cells arranged around a small lumen and tend to disappear at a short distance from the pyloric‐duodenal junction. The mucosal layer is characterised by leaf‐shaped villi and by crypts of Lieberkühn (McGraw‐Hill, 1966). The intestinal epithelium consists of two compartments: the proliferative crypts of Lieberkühn and long, finger‐shaped projections (villi); the latter contains a single columnar layer of fully differentiated epithelial cells.

**FIGURE 5:**
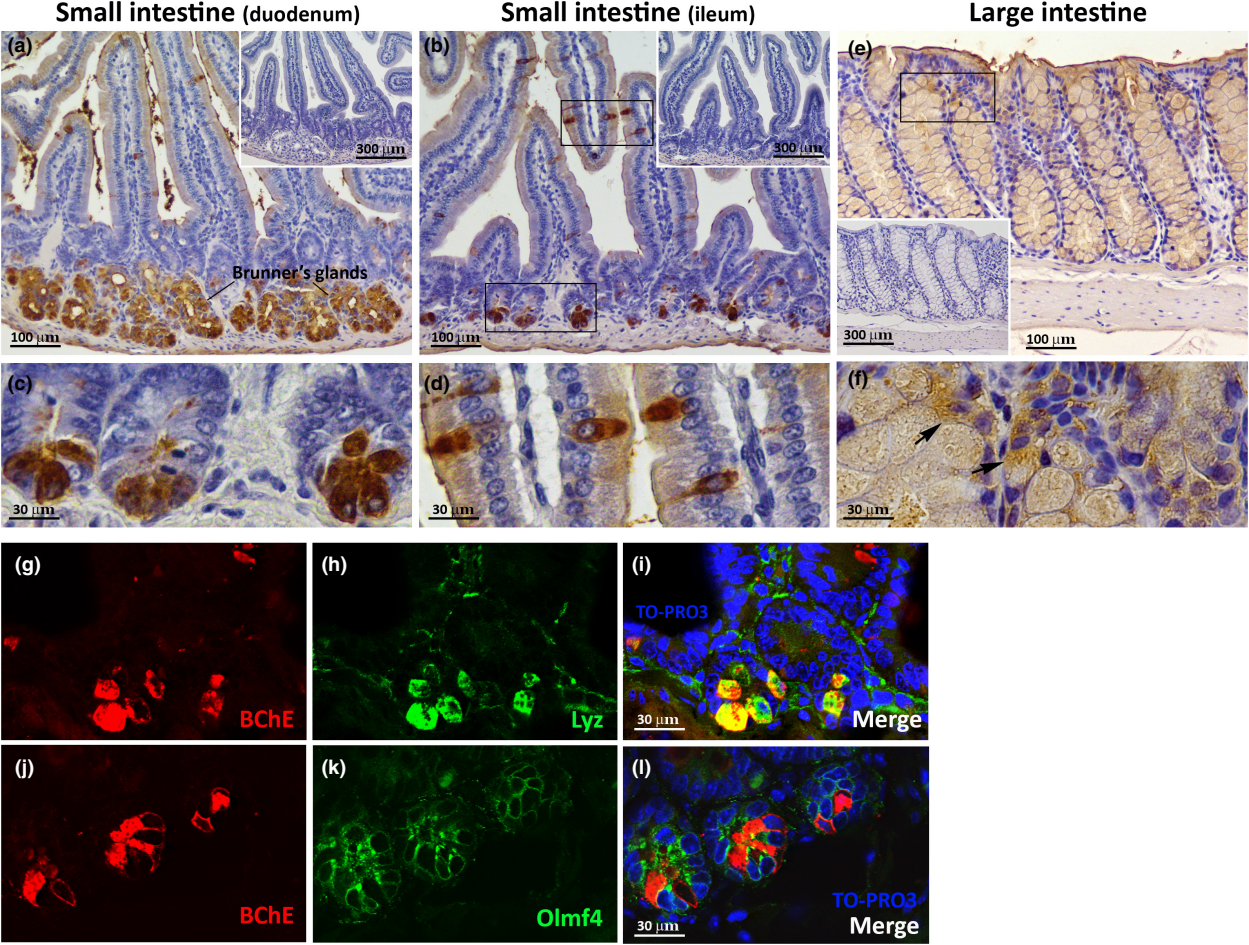
BChE distribution in the mouse small and large intestine. (a) Representative immunoperoxidase images showing BChE immunoreactivity in the duodenum. Specific BChE staining is visible along the villi, at the base of the crypts and in Brunner glands in the submucosal layer. Upper right corner in (a) negative control without the primary antibody. (b) Representative immunoperoxidase images showing BChE immunoreactivity in the ileum. Specific BChE staining is visible along the villi and at the base of the crypts. In (a) and (b), BChE staining is also visible near the tip of the villi in the intestinal lumen. Upper right corner in (b): negative control without the primary antibody. (c, d) Enlargements of the areas framed in (b) showing BChE^+^ cells in the intestinal crypts (c) and along the intestinal villi (d). (e) Representative immunoperoxidase images showing BChE immunoreactivity in the large intestine. Specific BChE staining is visible in the epithelial mucosal layer of the colon in clusters of goblet cells. Bottom left corner in (e): negative control without the primary antibody. (f) Enlargements of the area framed in (e) showing BChE^+^ cells resembling goblet mucous cells. (g–i) Representative confocal images of the intestinal crypts showing BChE^+^ cells (red) that also express lysozyme, a widely used marker of mouse Paneth cells (green). (j–l) Representative confocal images of the intestinal crypts showing BChE^+^ cells (red) that are negative for, but in close proximity to, cells positive for Olmf4, a widely used marker of mouse intestinal stem cells (green).

In duodenal samples, BChE^+^ cells were irregularly distributed along the villi, at the base of the crypts and in Brunner glands in the submucosal layer (Figure 5a). In the ileum, scattered BChE^+^ cells were identified along the villi and at the base of the crypts (Figure 5b–d). BChE staining was also detected near the tip of the villi, suggesting the presence of BChE in the intestinal lumen, where it may be secreted by enterocytes and/or crypt cells (Figure 5a,b).

Double‐label experiments, conducted to identify BChE^+^ cells in the crypts, showed that BChE^+^ cells at the crypt bottom also contained lysozyme (Figure 5g–i), the marker commonly used to identify Paneth cells (Clevers & Bevins, 2013).

Given the presence of BChE^+^ cells in the crypts and the fact that crypts contain functional stem cells scattered amongst Paneth cells, double‐label experiments were also performed to test the expression of olfactomedin‐4 (Olfm4), which has recently emerged as a robust marker of murine intestinal stem cells (Schuijers et al., 2014).

Although BChE^+^ cells were detected in close proximity to Olfm4^+^ cells, thus suggesting a role for BChE in stem cell physiology, none expressed both markers (Figure 5j–l).

### 3.5 BChE distribution in the large intestine

The mouse colonic epithelium consists of the surface epithelium (columnar and goblet cells) and the crypts of Lieberkühn (McGraw‐Hill, 1966). The mucosal wall is thick, with a thin muscularis mucosa and no discernible submucosa (Nguyen et al., 2015). Our immunohistochemical experiments documented BChE^+^ cells in the epithelial mucosal layer in clusters of goblet cells (Figure 5e,f). Intense BChE staining was detected in the cytoplasm of some cells (arrows, Figure 5f), whereas in most cells only the mucous content seemed to show BChE positivity. These data suggest that BChE is secreted by goblet cells, like mucins and other factors that are released into the intestinal lumen.

### 3.6 Proximity of BChE ^+^ cells to the proliferative niche in the mouse GI tract

**FIGURE 6:**
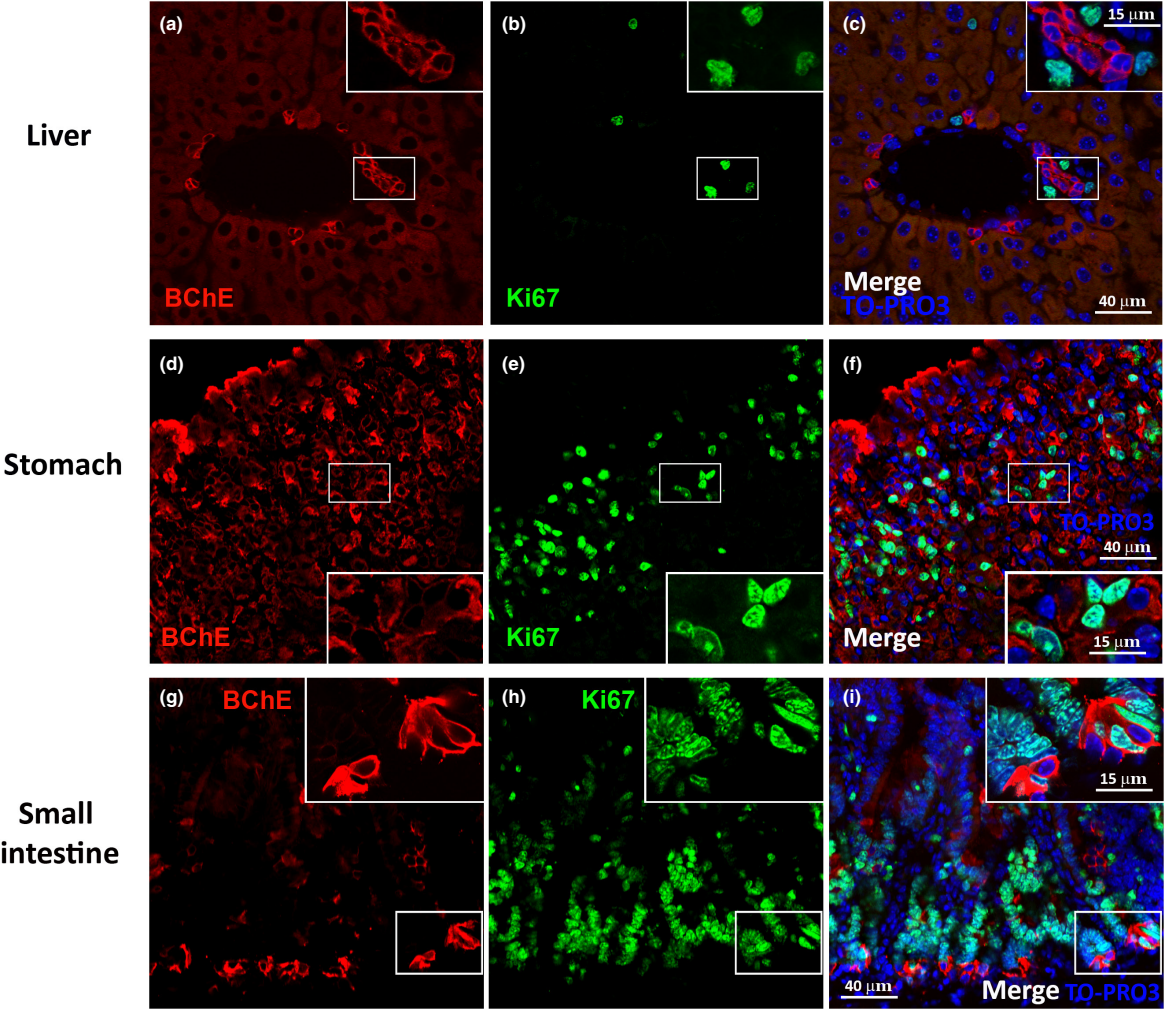
Close proximity of BChE^+^ cells to the proliferative niche in the mouse GI tract. (a–c) Representative confocal microscopy images of the liver showing BChE^+^ cells (red) that are negative for, but in close proximity to, cells positive for Ki67, a widely used marker of mouse proliferative cells (green). (d–f) Representative confocal images of the stomach showing BChE^+^ cells (red) negative for, but in close proximity to, Ki67^+^ cells. (g–i) Representative confocal images of the small intestine showing BChE^+^ cells (red) that are negative for, but in close proximity to, Ki67^+^ cells. Upper right corner of (a–i) enlargement of the areas framed in each panel.

To establish whether BChE^+^ cells are found near progenitor cells, we examined the distribution of Ki67^+^ proliferative cells. In the liver, BChE^+^ cells resembling bile duct epithelial cells were often found near Ki67^+^ cells (Figure 6a–c). Notably, in mice, the hepatic stem cell niche has been described in the canals of Hering and the space of Disse (Kordes & Häussinger, 2013). In the stomach, the proliferative zone is in the isthmus, a region between the gland and the pit (Barker et al., 2010), where BChE^+^ cells were detected near Ki67^+^ cells (Figure 6d–f). In the intestine, progenitor cells are found with Paneth cells in the crypts (Sato et al., 2011). Interestingly, BChE^+^ Paneth cells showed a close spatial relationship with Ki67^+^ cells in the crypts (Figure 6g–i). Collectively, BChE^+^ cells were present close to the proliferative niches of the liver, stomach and intestine.

## 4 DISCUSSION

BChE is a hydrolytic enzyme of the carboxylesterase family that is found in all mammals. Although a clear physiological function has not been identified for it, abnormal BChE serum levels are found in patients with conditions such as neurological disorders (especially Alzheimer's disease), obesity, type 2 diabetes and elevated cardiovascular risk (Darvesh et al., 2003). Interestingly, BChE hydrolyses the appetite‐promoting hormone ghrelin (Chen et al., 2015).

The bulk of BChE is synthesised in the liver and secreted into the circulation; however, a fraction is also expressed in numerous neuronal and non‐neuronal cells, especially cells with a barrier function such as epithelial and immune cells (Wessler et al., 2003). With regard to BChE distribution, most studies have investigated cholinergic enzyme activity by biochemical assays aimed at detecting the cells that are capable of producing and releasing it. They have found that BChE activity exceeds AChE activity in mouse liver, intestine, heart, lung and serum (Li et al., 2000). However, these investigations, which have quantified total BChE by homogenisation and enzyme isolation procedures, may have underestimated its levels in peripheral organs and may have been unable to identify its cellular localisation with precision (Darvesh et al., 2003). Notably, BChE distribution in the GI tract, where it would be able to perform several of its putative metabolic functions, has never been investigated by morphological techniques.

The present study was devised to examine BChE distribution in the mouse GI tract by immunohistochemistry and double‐label confocal microscopy. To validate the antibody, we investigated BChE presence and distribution in the GI tract and its liver expression in mice fed an HFD (Chen et al., 2017). By immunohistochemistry, BChE^+^ cells were identified in various GI regions and cell types: in the liver (both in hepatocytes and in cholangiocytes); in the keratinised epithelium of the oesophagus and forestomach, in the glandular portion of the stomach; in mucus‐secreting cells of duodenal Brunner glands and small and large intestinal epithelial cells. BChE^+^ luminal material was detected in the apical portion of the mucosa throughout the GI tract. Interestingly, in sections from the oesophagus, stomach and intestine, the smooth muscle cells of the muscularis mucosa expressed BChE, whereas the skeletal muscle cells of the muscularis externa were BChE‐negative. Although a few studies have examined the presence and activity of BChE in rodents as well as human skeletal muscle (Jbilo et al., 1994; Massoulié & Bon, 1982), they have not used morphological techniques.

The abundance of BChE^+^ cells documented in the mouse GI tract raises a number of questions about its physiological function(s).

To the best of our knowledge, this is the first study describing BChE in cholangiocytes. Interestingly, since ACh induces HCO3^−^ secretion and modulates bile formation and modification in the liver through activation of muscarinic receptors on the cholangiocyte basolateral plasma membrane (Tabibian et al., 2013), BChE produced by cholangiocytes may have a role in bile formation. As cholangiocytes are also part of the hepatic stem cell niche (Kordes & Häussinger, 2013), BChE may conceivably play a role in liver homeostasis and cell turnover. Notably, BChE molecules possess an HNK‐1 sugar epitope considered as an adhesive binding site, which plays a role in cell mobility during embryonic development (L'Hermite et al., 1996); accordingly, we also detected BChE^+^ cells close to Ki67^+^ cells in other stem cell niches, such as the isthmus of the stomach and the intestinal crypts (Barker et al., 2010; Sato et al., 2011). Since in the gut endogenous ACh released from the intestinal epithelium is involved in cell renewal via nicotinic receptors in Paneth cells (Takahashi et al., 2018), the presence of BChE in these cells could indicate its involvement in the renewal processes of intestinal mucosal cells.

Our experiments also revealed a spatial relationship between ghrelin‐producing cells and BChE^+^ parietal cells in the oxyntic mucosa of the stomach. The role of BChE in hydrolysing the hunger hormone ghrelin (Chen et al., 2017; De Vriese et al., 2004) connects it with obesity and appetite regulation. Notably, human studies have disclosed that serum BChE activity correlates with serum triglyceride, total cholesterol and insulin levels, pointing to the involvement of BChE in fat metabolism (Randell et al., 2005; Santarpia et al., 2013). Since the stomach is the main source of ghrelin, our findings suggest that BChE plays a paracrine action on ghrelin in the oxyntic mucosa.

Furthermore, the presence of BChE in epithelial secretory cells that are in direct contact with the gastric and intestinal lumina and with other cells inside the alimentary canal suggests that the enzyme exerts a direct action on luminal content. The ubiquitous distribution of a bioscavenger such as BChE along the GI tract, documented in this study, and its presence in “the major organs of entry”, such as lung, liver and serum (Jbilo et al., 1994), suggest a role for it as a detoxifier, acting as a first‐line barrier against compounds introduced with the diet. The notion is consistent with the hypothesis that it acts as a detoxification enzyme, which Ziegler (Ziegler, 1991) based on three criteria: the presence of BChE in the main organs of entry, its broad substrate range and its different concentrations in different species in relation to their diets (Johnson & Moore, 2012). Notably, the large acyl pocket of BChE accommodates a wide range of substrates like traditional organophosphates and carbamates as well as compounds such as cocaine (Xie et al., 1999) and heroin (Qiao et al., 2014). Interestingly, numerous BChE substrates derive from natural plant metabolism and are used by the plants themselves as protection against herbivores (Johnson & Moore, 2012).

Lastly, foods are natural sources of substances that may exert crucial effects on the nervous system, such as neurotransmitters (Briguglio et al., 2018). In particular, ACh is found in ingested meat as well as in more than 50 plant species belonging to all the major systematic groups, whilst *Lactobacillus* species in the gut microbiota have been demonstrated to produce ACh (Lyte, 2011; Smallman & Maneckjee, 1981). Therefore, BChE might act as a detoxifier against ACh released into the gastric and intestinal lumina in response to food ingestion. Accordingly, BChE released from gastric and intestinal secretory cells by exocrine processes or from oesophageal endothelial cells would be able to exert a direct action on the concentration of this powerful molecule in the GI lumen.

Altogether, the ubiquitous distribution of BChE along the mouse GI tract, emerging from our study, suggests that it is involved in a wide range of major physiological functions. These functions, which include bile formation and liver homeostasis, appetite and fat regulation, mucosal renewal processes and resistance to GI toxicity, warrant further detailed investigation with obesity and calorie restriction mouse models and *in vitro* culture systems that mimic the effects of BChE treatment as well as analysis of human samples from donors with normal and high and low body mass index.

## AUTHOR CONTRIBUTIONS

IS designed the study, performed experiments, data analysis and interpretation and wrote the manuscript. SA, JP, EDM and MS performed experiments, data analysis and interpretation. AG designed the study, provided lab facilities and critically revised the manuscript.

## CONFLICTS OF INTEREST

The authors declare no conflict of interest.

## Supporting information


Figure S1

## Data Availability

The data that support the findings of this study are available from the corresponding author upon reasonable request.
