# Supplementary figures and images for: Butyrylcholinesterase distribution in the mouse gastrointestinal tract: An immunohistochemical study
Source: J Anat. 2022 Aug 25;242(2):245–56. doi: 10.1111/joa.13754 (PMC9877478; doi:10.1111/joa.13754)

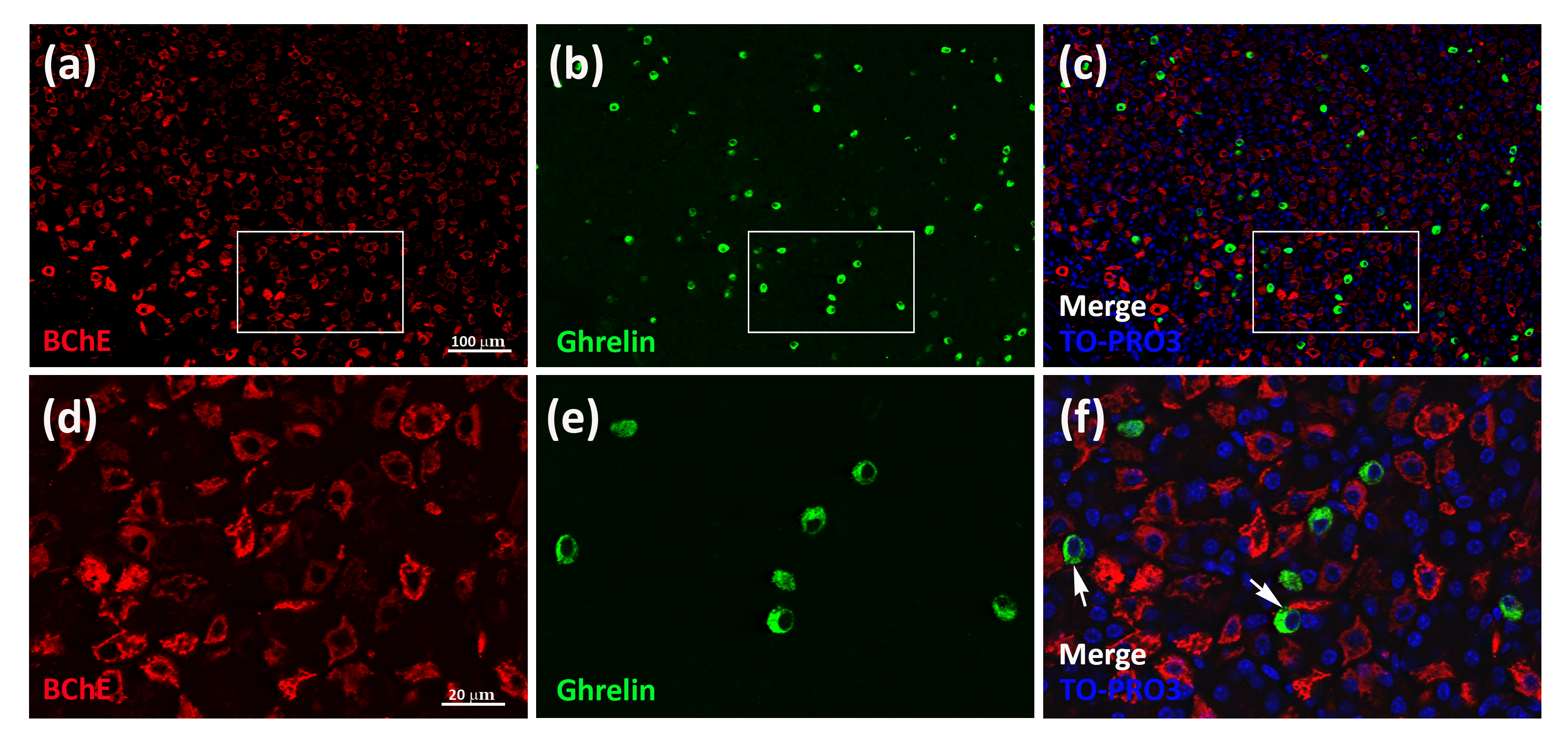

Supplement: Supplementary file 1 — Figure S1 [file JOA-242-245-s001.tif]
